# Implications of intratumoral microbiota in tumor metastasis: a special perspective of microorganisms in tumorigenesis and clinical therapeutics

**DOI:** 10.3389/fimmu.2025.1526589

**Published:** 2025-02-10

**Authors:** Lei Zhang, Xichu Duan, Yanhua Zhao, Dejiu Zhang, Yuan Zhang

**Affiliations:** Institute for Translational Medicine, The Affiliated Hospital of Qingdao University, Qingdao University, Qingdao, China

**Keywords:** tumor metastasis, intratumoral microbiota, tumor pathogenesis, mechanisms, clinical perspective

## Abstract

Tumor metastasis is the main cause of therapeutic failure and mortality in cancer patients. The intricate metastastic process is influenced by both the intrinsic properties of tumor cells and extrinsic factors, such as microorganisms. Notably, some microbiota have been discovered to colonize tumor tissues, collectively known as intratumoral microbiota. Intratumoral microbiota can modulate tumor progression through multiple mechanisms, including regulating immune responses, inducing genomic instability and gene mutations, altering metabolic pathways, controlling epigenetic pathways, and disrupting cancer-related signaling pathways. Furthermore, intratumoral microbiota have been shown to directly impact tumor metastasis by regulating cell adhesion, stem cell plasticity and stemness, mechanical stresses and the epithelial-mesenchymal transition. Indirectly, they may affect tumor metastasis by modulating the host immune system and the tumor microenvironment. These recent findings have reshaped our understanding of the relationship between microorganims and the metastatic process. In this review, we comprehensively summarize the existing knowledge on tumor metastasis and elaborate on the properties, origins and carcinogenic mechanisms of intratumoral microbiota. Moreover, we explore the roles of intratumoral microbiota in tumor metastasis and discuss their clinical implications. Ongoing research in this field will establish a solid foundation for novel therapeutic strategies and clinical treatments for various tumors.

## Introduction

**Figure 1:**
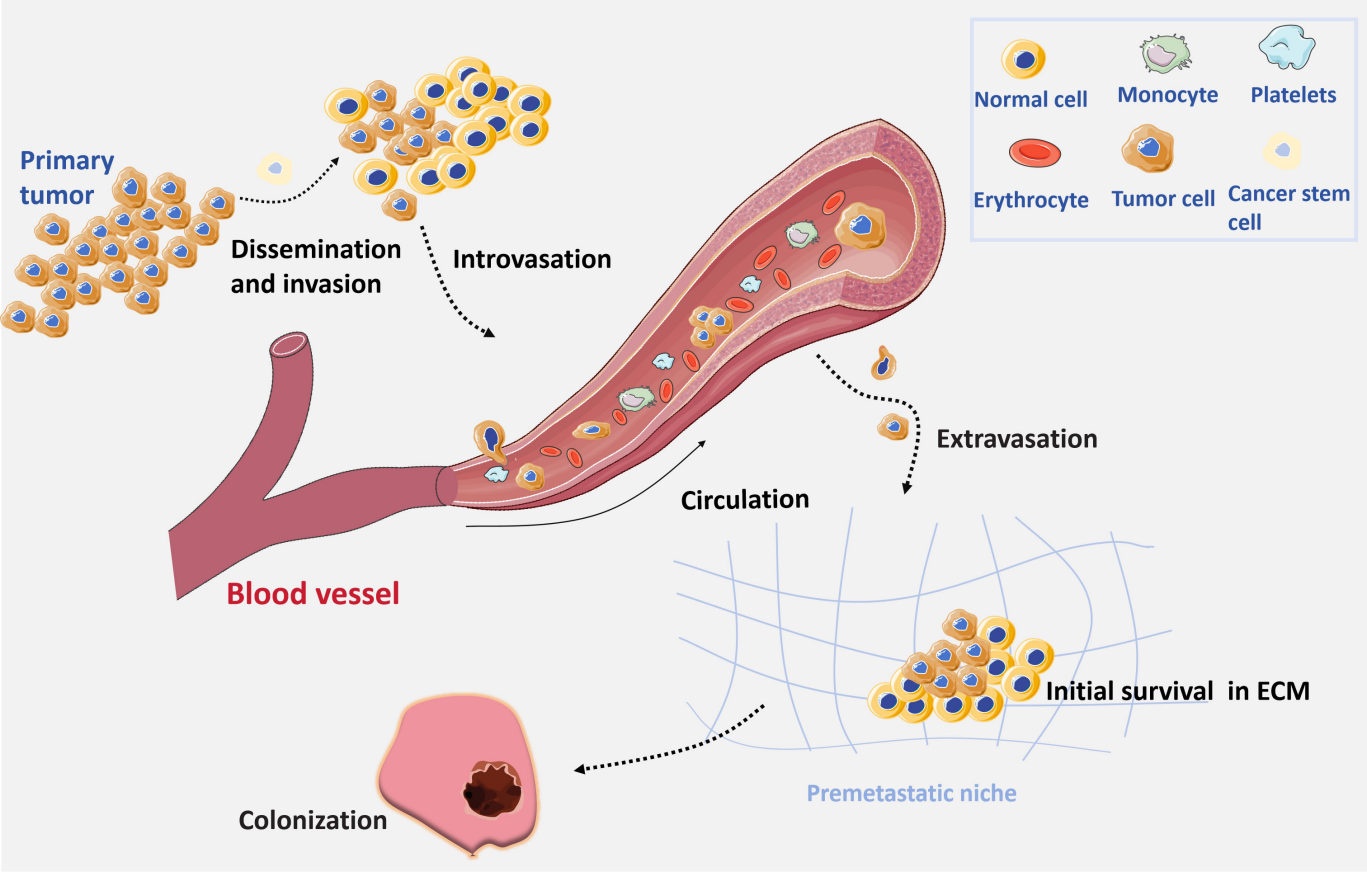
Tumor metastasis processes. In the initiation, primary tumor cells undergo EMT and penetrate surrounding tissues (Invasion and dissemination), which allow tumor cells to disseminate into nearby tissue. Then tumor cells break into lymphatic vessels or blood vessels (Intravasation) and most of them die because of the physical stresses and immune surveillance. The surviving tumor cells extravasate through the circulatory vascular walls into distal tissues (Extravasation), adapt to new microenvironment and colonize distal tissues (Colonization).

Tumor metastasis is a main cause of therapeutic failure in oncology. Malignant tumors are inherently prone to metastasize, whereas benign tumors do not exhibit this characteristic (1). Tumor metastasis consists of complex multistep processes through which malignant cells disseminate from a primary site through lymphatic channels, blood vessels, or body cavities, ultimately reaching a distal site and establishing secondary tumors. In general, there are several necessary steps of tumor metastasis (1) (**Figure 1**): invasion, dissemination, intravasation, extravasation, and colonization. (1) Invasion and dissemination: primary tumor cells undergo epithelial-mesenchymal transition (EMT) and penetrate surrounding tissues through breaching the basement membrane. These events allow tumor cells to disseminate into adjacent tissues (2). (2) Intravasation: Tumor cells break into lymphatic vessels or blood vessels and survive in the circulatory period. Most tumor cells in circulation succumb to physical stresses and immune surveillance. Therefore, only a small portion of these circulating tumor cells survive to progress to the subsequent stage of metastasis (3). (3) Extravasation: The surviving tumor cells extravasate through the circulatory vascular walls of blood or lymphatic vessels into distal tissues. (4) Colonization: tumor cells adapt to new microenvironments, colonize distal tissues, proliferate and spread in new locations, thereby establishing new metastatic tumors (3). Malignant tumor metastasis typically take place in four principal pathways: directly extension to adjacent tissues, lymphatic spread, hematogenous dissemination, and implantation within body cavities (2, 3). The intricate processes are influenced by complicated intrinsic properties of tumor cell, such as EMT status, genetic and epigenetic alteration, chromosome stability, and metabolic adaptations (4). Additionally, some external factors are also involved in metastasis, such as immune responses, the composition of extracellular matrix (ECM), and gut microbiota (5).

The relationship between microorganisms and human health is a hot topic and substantial advancements have been made in understanding these complex interactions. Microorganisms exist in various human tissues, and their symbiotic relationships with the host affects human physiology and disease states (6). The gut microbiota, perhaps the most thoroughly studied, plays pivotal roles in multiple physiological and pathological processes, including digestion, immune reaction, cardiovascular health, tumor development, and so on (2). Except for the digestive tract and skin known to contain diverse microbial communities, other tissues, both healthy and malignant, were traditionally believed to have no microorganisms. However, in the past five years, emerging studies have challenged this speculation by identifying microorganisms in various tumor tissues (7–9). This unique microbial community, named intratumoral microbiota, has aroused significant interest among researchers. Intratumoral microbiota have now been found in over 33 different types of cancers (10–12), highlighting its widespread presence and potential significance in tumor development. Intratumoral microbiota reside within tumor tissue, are embedded in the tumor microenvironment, and can also be found within the surrounding stroma. This complex community comprises diverse species of bacteria, fungi and viruses (10). Intratumoral microbiota may contribute to tumor development, metastasis, progression, and treatment via various mechanisms, such as modulating inflammation, angiogenesis, and immune surveillance, or even by affecting the metabolic environment of the tumor (5).

The current findings have demonstrated that intratumoral microbiota play a significant role in modulating tumor development. However, compared with the growing number of studies on gut microbiota, our knowledge of these microbial communities within tumor is still immature and limited. The challenge lies in accurately and specifically manipulating intratumoral microbiota without disrupting the balance of the microbiota elsewhere in the body. This is a significant obstacle in both tumor metastasis research and the advancement of clinical treatments. A comprehensive and in-depth understanding on the composition, functions, and clinical implications of intratumoral microbiota is paramount. Additionally, adopting a forward-looking perspective on future development is of great significance to inform the development of novel diagnostic and therapeutic strategies in oncology. In this review, we summarize existing knowledge regarding on the properties and potential action mechanisms of intratumoral microbiota in tumor pathogenesis, with a particular emphasis on their involvement in tumor metastasis. Furthermore, we consolidate the current findings on the regulatory role of intratumoral microbiota in tumor metastasis and explore the possible clinical applications of intratumoral microbiota in tumor diagnostics and therapeutic approaches. This review will offer a comprehensive understanding for further research and lay a foundation for clinical applications of intratumoral microbiota.

## Characteristic of intratumoral microbiota

### Discovery and properties

The recognition of microorganisms within tumors can be traced back to the 20th century. In 1907, Doyen et al. isolated and identified a bacterium from three cases of malignant tumors and confirmed its tumorigenic capacity (13). Nevertheless, despite this breakthrough, these findings were questioned and not widely accepted. This skepticism was largely due to the limitations of the experimental conditions available at the time, particularly the challenge of maintaining an aseptic environment. In 1911, Rouse indicated that a virus extracted from avian sarcoma, later named the *Rous sarcoma* virus, could induce tumor occurrence. This provided the first experimental evidence linking viruses to the development of cancer (14). Further progress came in 1964 when Epstein and Barr identified the *Epstein-Barr virus* (*EBV*), the first discovered human virus within Burkitt’s lymphoma (15). Subsequent studies have expanded our understanding of the involvement of various tumor-resided microbiota in cancer development (10, 16). Some of these microorganisms have shown potential as diagnostic and prognostic biomarkers (10, 16), indicating their possible clinical relevance. For example, the bacterium *Helicobacter pylori* (*H. pylori)* is closely related to the formation of gastric cancer (17, 18), with multiple studies demonstrating a causal relationship. Intratumoral microbiota possess unique properties that facilitate its tumor-related functions.

First, heterogeneity and complexity. They exhibit high heterogeneity, diversity and complexity (10). Their composition may be influenced by various factors such as genetic background, environmental conditions, and lifestyle and dietary structure of the hosts. Based on intracellular and extracellular localization within tumor tissues, intratumoral microbiota can be divided into intracellular tumor-resident microbiota (InTM) and extracellular tumor-resident microbiota (ExTM) (19, 20). These subcategories may have different functions and effects on tumor progression. Second, symbiosis with tumors (19, 20). Unlike other microorganisms that typically colonize body surfaces or reside within the gastrointestinal tract, these microbes predominantly parasitize in tumor tissues, and some may even enter the interior of tumor cells. This specialized localization implies an adaptation to the unique tumor microenvironment. Third, promoting microecosystem formation (11). Intratumoral microbiota generate a special microbial ecosystem in tumor tissues. This ecosystem engages in complex interactions with host cells, which potentially affects the composition and stability of the tumor microenvironment. This may subsequently influence immune response, angiogenesis, and tumor metastasis. Fourth, association with tumor therapy (21, 22). Intratumoral microbiota have the potential to impact drug metabolism and decomposition, which in turn influence the host’s response to anti-tumor treatments, including chemotherapy and immunotherapy (22). Delving into the unique characteristics of intratumoral microbiota can significantly enhance our understanding of tumorigenesis and help develop novel therapeutic strategies.

### Advanced techniques for intratumoral microbiota study

Over a century ago, doctor William Coley proposed that every type of malignant tumors maybe influenced by the involvement of microorganisms (23). However, due to technological barriers, no evidence can confirm this hypothesis until recent decades. With the advancement of next-generation sequencing (NGS) techniques, specifically 16S ribosomal DNA sequencing, it has become possible to distinguish and analyze bacterial DNA within tumors, thereby confirming the presence of bacteria in tumors (21, 24). Despite these advances, the subsequent findings were hindered by the low abundance of intratumoral microbiota, host genome contamination and environmental noise signal, as well as many other factors. These obstacles lead to poor consistency and make it difficult to draw definitive conclusions (25, 26). Nonetheless, advancements in technology and methodology have helped to overcome these barriers. Nejman et al. employed an optimized multiregional 16S sequencing method to analyze the microbial composition in over 1500 tumor tissues from seven cancer types (10). They reported that each cancer type has a unique microbial signature, with breast cancer showing a particularly rich and diverse microbial community (10). Moreover, Fu et al. further validated these findings by using a two-step 16S enrichment sequencing technique, confirming a microbial composition in breast cancer (27). In addition, whole-genome and whole-transcriptome analyses have emerged as powerful tools in the study of tumor-specific microbial profiles.

By bioinformatics comparisons of microbial data, it has become possible to distinguish between healthy individuals and cancer patients (12, 21), suggesting the diagnostic potential of microbial profiles. These innovative methods and techniques have greatly advanced the identification and detection of intratumoral microbiota in both murine and human cancer models. However, this field still requires more effective and refined methods to further advance the research.

### Origin of the intratumoral microbiota

Although there is increasing research on the functions of intratumoral microbiota, their origins remain uncertain. Understanding the origins of intratumoral microbiota is important for developing targeted therapies and preventive strategies. By illustrating the pathways through which these microorganisms infiltrate and proliferate in tumor tissues, researchers can develop innovative strategies to suppress their colonization, potentially improving cancer treatment outcomes. Several possible origins and pathways have been proposed the origins of intratumoral microbiota.

(1) Blood circulatory system invasion. Microbiota from other parts of the body, such as the oral cavity and intestinal tract, may enter the bloodstream and penetrates blood vessels into tumors (7, 28). Experiments in mice have shown that intravenous injection of *Fusobacterium nucleatum (F. nucleatum)* can ultimately lead to colonization of colon tumor, indicating that *F. nucleatum* can reach tumor sites through the circulatory system (29). Similarly, another study conducted in mouse mammary tumors further verified *F. nucleatum* can traverse the blood circulatory system to colonize tumor sites (7).

(2) Mucosal tissue invasion. Microorganisms that colonize the mucosal organs, such as lung, colon, esophagus and cervix can infiltrate tumors via the damaged mucosa (30, 31). Except for mucosal organs, even non-mucosal organs like the pancreas may obtain intratumoral microbiota through the migration of gut bacteria when the mucosal barrier is impaired (32).

(3) Adjacent tissue invasion. Intratumoral microbiota may also originate from microbial communities in neighboring normal tissues. Studies have shown a similarity between microbiome communities of adjacent normal tissues and tumor tissues (33, 34). In addition, virus or bacterial infections, along with the chronic inflammation they trigger, may eventually contribute to tumor development (30, 35). Therefore, it is possible that microbiota from surrounding normal tissues can infiltrate tumor tissues. Nonetheless, this hypothesis still needs further investigation and confirmation.

(4) Tumor microenvironment assistance. The unique characteristics of the tumor microenvironments such as hypoxic, immunosuppressive, and a nutrient-enriched metabolic environment, can promote microbiota colonization (36). These conditions may create a proper environment for microorganisms to grow within tumors. More research should be conducted to explore these factors and to elucidate the mechanisms by which they facilitate the colonization and growth of intratumoral microbiota.

### Intratumoral microbiota in tumor metastasis

Based on the advanced techniques, the subsequent findings have confirmed the presence of intratumoral microbiota. Many of these microorganisms are shown to be linked to tumor metastasis (**Table 1**). Eun and colleagues explored the microbial profile in Oral squamous cell carcinoma (OSCC) patients with lymph node (LN) metastasis (41).They found that certain bacteria, such as *Fusobacterium, Tannerella Prevotella, Stomatobaculum, Bifdobacterium, Peptostreptococcaceae, Shuttleworthia*, and *Finegoldia*, displayed altered abundance in patients with LN metastasis (41). Notably, *Prevotella, Stomatobaculum, Bifdobacterium and Fusobacterium* exhibited the most significant differences between patients with and without metastasis (41). In patients with esophageal squamous cell carcinoma (ESCC), an evident increase in *F. nucleatum* levels was observed, implying its potential involvement in metastasis (40). *F. nucleatum*, a widely studied Gram-negative anaerobic bacterium, has been identified as an enriched oncogenic bacterium, contributing to tumor progression in various cancer types (42–44). Its role in colorectal cancer (CRC) development and metastasis has been extensively studied (43, 44). Zhang et al. demonstrated the important role of *F. nucleatum* in CRC metastasis (9). Furthermore, Guo et al. found that exosomes secreted by *F. nucleatum* could infect CRC cells, thereby promoting their metastatic processes (37). *F. nucleatum* is also implicated in metastatic progression of breast tumors (7). In certain murine tumor models, distinct bacterial communities such as *Staphylococcus*, *Enterococcus, Streptococcus, and Lactobacillus*, were identified as markers to distinguish normal tissues from breast or lung tumor tissues (27). These bacterial communities were associated with lung metastasis (27). Camilla Urbaniak and colleagues employed 16S rRNA gene sequencing on clinical breast tissues and discovered elevated abundances of *Bacillus, Enterobacteriaceae and Staphylococcus* in tumor tissues compared with normal tissues (38). *Pseudomonas genus* is involved in human wound infections, cystic fibrosis, sepsis and so on. *Pseudomonas aeruginosa* was detected in 16S rRNA sequencing studies and found to be related to distant metastases of breast cancers (39). *Bacteroides fragilis* (*B. fragilis*), a bacterium commonly resident in the colon, has been found to trigger breast cancer growth and metastasis (8). This finding indicates the profound impact of the microbiota from one body site on tumor behavior elsewhere, suggesting intricate interactions between these microbial communities and tumor biology. These studies collectively illustrate the known connections between intratumoral microbiota and tumor metastasis. The identification of additional intratumoral microbiota is anticipated to facilitate the advancement of novel therapeutic approaches targeting specific microorganisms or microbiota-related pathways.

**Table 1:** Intratumoral microbiota related to tumor metastasis.

Cancer type	Strains	Function	Reference
**Colorectal cancer**	Fusobacterium nucleatum	Enhance CRC cell metastasis by regulating the ALPK1-NF-κB-ICAM1 pathway.	(9)
		Induced functional exosomes to facilitate CRC metastasis	(37)
**Breast cancer**	Staphylococcus, Lactobacillus, Enterococcus, and Streptococcus	Inhibit RhoA-ROCK pathway, thereby disassembling stress fiber and decreasing contractile	(27)
	Bacillus, Enterobacteriaceae and Staphylococcus. Escherichia coli, Staphylococcus epidermidis	Induce DNA double-stranded breaks	(38)
	Pseudomonas genus	Impact breast cancer cell signaling and drug responsiveness.	(39)
	Fusobacterium nucleatum	Suppresses accumulation of tumor infiltrating T cells and promotes tumor growth and metastatic progression	(7)
	Bacteroides fragilis	Induces growth and metastatic progression of tumor cells by activation of the β-catenin and Notch1 pathways	(8)
**Esophageal squamous cell carcinoma**	Fusobacterium nucleatum	—–	(40)
**Oral squamous cell carcinoma**	Fusobacterium, Tannerella Prevotella, Stomatobaculum, Bifdobacterium, Peptostreptococcaceae, Shuttleworthia, Finegoldia	—–	(41)

### Regulatory mechanisms of intratumoral microbiota in tumor progression

**Figure 2:**
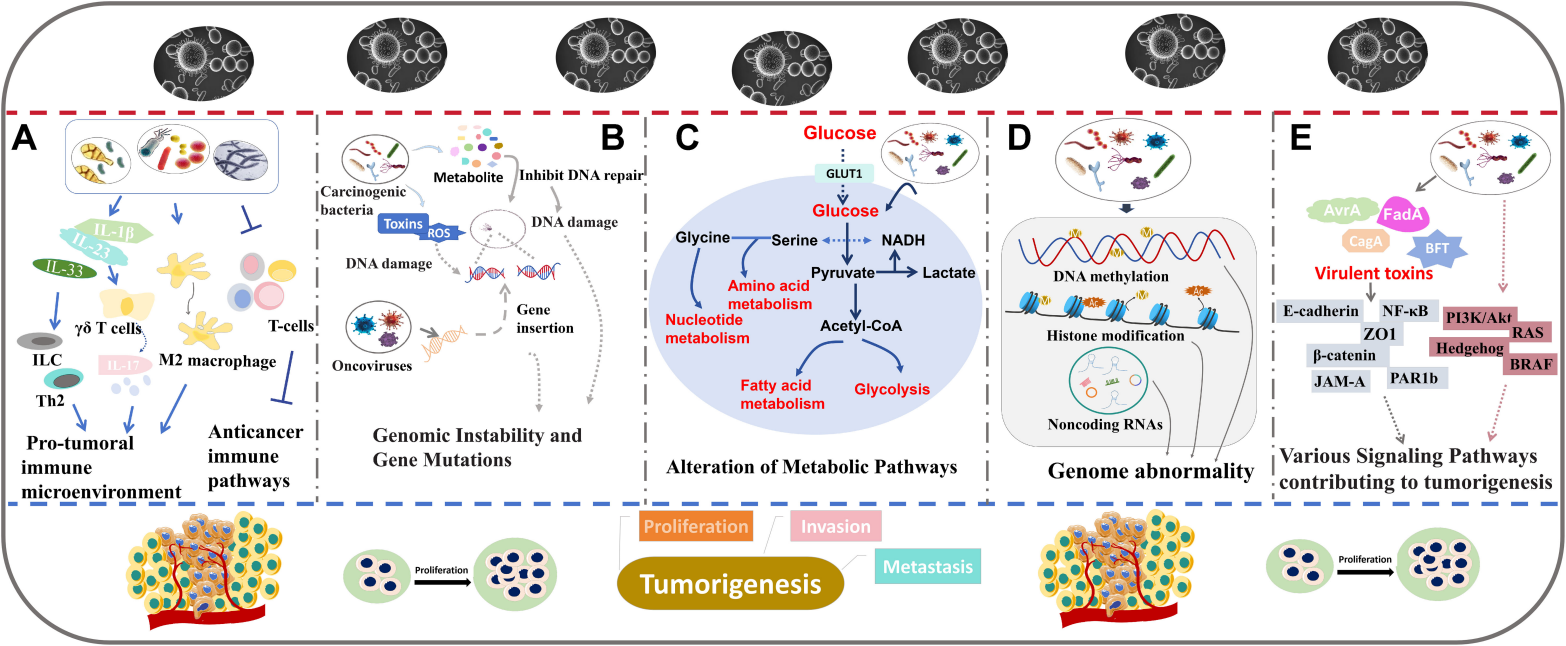
The role of intratumoral microbiota in tumor development. The major mechanisms including regulating immune responses **(A)**, inducing genomic instability and gene mutations **(B)**, altering metabolic pathways **(C)**, regulating epigenetic pathways **(D)**, and interfering with cancer-related signaling pathways **(E)**.

Gut microbiota, the most common and multifunctional microbial community in the body, can remotely regulate the occurrence and development of many tumors by producing metabolites and modulating the immune system. In contrast, intratumoral microbiota are located directly within tumor tissues or the tumor microenvironment. The close physical proximity to the tumor cells may allow them to influence tumor behavior in more diverse ways (**Figure 2**). Some potential mechanisms through which intratumoral microbiota may affect tumor progression are summarized as follows.

### Modulation of immune responses

Intratumoral microbiota may affect tumor progression by regulating the host’s immune response (**Figure 2A**). In particular, in CRC, *F. nucleatum* has been shown to disrupt immune system dynamics, potentially facilitating tumor development and progression (45). *F. nucleatum* can inhibit the aggregation of tumor stromal CD3^+^ lymphocytes and CD3^+^CD4^+^CD45RO^+^ cells, key components of T-cell-mediated immunity (45). Meanwhile, *F. nucleatum* promotes the accumulation of tumor-infiltrating myeloid cells, including CD11b^+^ myeloid cells and macrophages associated with tumors (45). Therefore, the suppressive effect of *F. nucleatum* on T-cell-mediated anti-tumor immune responses may enhance CRC development. In another study, the abundance of intratumoral *F. nucleatum* was found to be positively correlated with the ratio of CD163^+^ to CD68^+^ macrophages in microsatellite instability (MSI)-high CRCs (46). This change in macrophage ratio indicates an increased proportion of polarization towards the immune-suppressive M2 phenotype. In addition, high intratumoral *F. nucleatum* levels suggest a reduced presence of FoxP3+ cells, which are mainly pro-inflammatory non-regulatory T (Treg) cells in MSI-high CRCs (46). Treg cells are correlated with tumor growth. Simone et al. have revealed that highly expression of some Treg cell-specific signature genes may predict poor overall survival in both non-small cell lung cancer and CRC patients (47). Another investigation showed that Treg cells with enriched cytokine thymic stromal lymphopoietin (TSLP) receptor could promote CRC progression (48). The decreased FoxP3+ cells and the increased M2-polarized macrophages facilitate the formation of pro-tumoral immune microenvironment, contributing to enhanced tumor growth and invasion (46). These findings suggest that the presence of *F. nucleatum* within CRC tumors can disrupt immune system equilibrium, thereby promoting pro-tumoral immune responses. Lowering the level of *F. nucleatum* can suppress CRC growth, indicating that targeting this bacterium may represent an effective therapeutic strategy (46). In pancreatic ductal adenocarcinoma (PDAC), the intratumoral fungal microbiome, such as *Malassezia* and *Alternaria*, has been shown to increase the level of interleukin-33 (IL-33) (49). IL-33 can recruit and activate immune-related T helper 2 cells and innate lymphoid cells (ILC), thereby creating an environment that enhances tumor progression. The activation of these cells can generate an immunosuppressive tumor microenvironment, promoting PDAC growth (49). Polymorphonuclear neutrophils (PMNs) affect the host’s acute response to infection. PMN depletion resulted in an increased abundance of *Akkermansia* and a decrease in *Proteobacteria* (50). PMN depletion can trigger an acute inflammatory response by inducing interleukin-17 (IL-17) expression, which is dependent on bacterial presence (50). This increase in IL-17 expression prompts the infiltration of intra-tumor B cell, thereby promoting colon tumor progression. These findings suggest that PMN deficiency may exert a pro-tumor effect by promoting inflammation and modifying the tumor microenvironment (50). In lung adenocarcinoma (LUAD), commensal bacteria including *Staphylococcus*, *Streptococcus*, *Lactobacillus*, and members of the *Pasteurellaceae* family have been revealed to stimulate the production of Myd88-dependent interleukin-1β (IL-1β) and interleukin-23 (IL-23) (24). Subsequently, this leads to the activation of γδ T cells capable of secreting IL-17 and other pro-inflammatory effectors. This process may intensify inflammatory activity and dysbiosis, consequently driving LUAD progression (24). Intratumoral *Pasteurella* has been found to be positively correlated to cytotoxic CD8^+^ tumor-infiltrating T lymphocytes (TILs) and negatively associated with M2-like macrophages, indicating a potentially enhanced immune response capable of inhibiting tumor growth (51). *Coriobacteriaceae* shows an opposite immune response (51). These immune activities are involved in the lung tumorigenesis. Elevated *Pasteurella* levels may inhibit lung tumor cell growth, while an abundance of *Coriobacteriaceae* might promote lung tumor cell growth (51).These observations indicate that intratumoral microbiota can significantly affect tumor progression and treatment outcomes by modulating immune pathways.

### Induction of genomic instability and gene mutations

Intratumoral microbiota such as oncoviruses and carcinogenic bacteria, may directly induce tumor formation by affecting genomic instability and promoting gene mutations (**Figure 2B**). The underlying mechanisms are diverse, predominantly involving the integration of microbial genomes into host chromosomes or the production of genotoxic substances that cause DNA damage, ultimately leading to tumorigenesis.

Oncoviruses may be responsible for the formation of more than 10% of human malignancies (52), with *Hepatitis B Virus* (HBV) and human papilloma virus being the most recognized examples. They can induce tumors by inserting their own genetic material into the host genome, thereby disrupting physiological processes and cell cycle of hosts (53, 54). This can lead to cellular transformation and malignancy. For example, the integration of HBV DNA into primary human hepatocytes has been shown to destroy normal gene regulation pathways, causing hepatocellular carcinoma (HCC) (55, 56). *Human T-lymphotropic virus type 1* (HTLV-1), a retrovirus, can impair DNA repair mechanisms, leading to genomic instability and an accumulation of oncogenic mutation, which in turn elevates the risk of adult T-cell leukemia (57).

Many carcinogenic bacteria are identified to trigger tumor formation by causing DNA damage. *F. nucleatum* secrets an adhesion to activate the E-cadherin/β-catenin pathway which increase checkpoint kinase 2 activity, leading to DNA damage in CRC cells (58). Another study found that *F. nucleatum* infection promotes the formation of DNA double-strand breaks (DSBs) via the Ku70/p53 pathway, thereby initiating OSCC development (59). Similarly, *Escherichia coli* (*E. coli*) and *Staphylococcus* strains extracted from breast tumor tissues were revealed to cause abnormal DSBs in Hela cells (38). Analysis of intestinal mucosa in patients with familial adenomatosis reveals an increased abundance of both *E. coli* and *Bacteroides fragilis* (*B. fragilis*). Mechanism exploration shows that their colonization within epithelial cells triggers substantial DNA damage by stimulating interleukin-17 (IL-17) production (60). *Enterotoxigenic B. fragilis* (*ETBF*) and pks^+^ *E. coli* can produce toxins that cause DNA damage, potentially leading to tumor initiation in the colon (60, 61). *Porphyromonas gingivalis* (*P. gingivalis*) and *Streptococcus anginosus* (*S. anginosus*) can enhance DNA damage and facilitate the onset of oral carcinogenesis by metabolizing ethanol into acetaldehyde (62, 63). This conversion results in the generation of DNA adducts and the inhibition of DNA repair enzyme activity (62, 63).

### Alteration of metabolic pathways

Some microorganisms may influence tumor growth and metastasis by disrupting the metabolic pathways of host cells such as modifications to energy metabolism, amino acid metabolism, and other vital biological processes (**Figure 2C**).

*F. nucleatum* has been shown to disrupt host metabolism. *F. nucleatum* accumulation in OSCC tissues has been observed to upregulate glucose transporter 1 (GLUT1) and to promote lactate deposition by activating GalNAc Autophagy-TBC1D5 pathway. This increased lactate deposition leads to a more acidic tumor microenvironment, facilitating tumor progression and metastasis (64). *Kaposi’s sarcoma associated herpesvirus* (*KSHV*), a known cause of Kaposi’s sarcoma, has been found to stabilize hypoxia-inducible factor 1 alpha (HIF1α), a key regulator of cellular response to hypoxia. HIF1α stabilization leads to upregulation of the genes involved in the glycolysis metabolic pathway (65), signifying a metabolic switch from aerobic cycle to glycolysis even under oxygen-rich conditions. This metabolic reprogramming promotes tumor development in the hypoxic tumor microenvironment, a phenomenon known as the Warburg effect (65). *Merkel Cell Polyomavirus* (MCPyV), the etiological agent of Merkel cell carcinoma, produces an oncoprotein referred to as MCPyV small tumor antigen (ST). MCPyV ST upregulates the monocarboxylate lactate transporter (MCT1), a glycolytic gene, thereby promoting aerobic glycolysis (66). Hieken and colleagues identified an altered microbial composition in breast cancer tissues, with specific bacteria shown to modulate metabolic pathways. *Fusobacterium, Atopobium, Hydrogenophaga, Gluconacetobacter and Lactobacillus* can reduce inositol phosphate metabolism (67), which may influence cellular growth and signal transduction. Moreover, *Helicobacter* and *Lactobacillus* can regulate various metabolism pathway, including those of amino acids, nucleotides, and glycerophospholipids (68). The changes can contribute to tumor progression. In cervical cancer, a microbiota composition devoid of *Lactobacillus* such as *Atopobium*, *Gardnerella*, *Streptococcus* and *Prevotella* can disturb amino acid and nucleotide metabolism (69). This disruption affects cellular homeostasis and promote cervical cancer development.

### Regulating epigenetic pathways

Intratumoral microbiota is capable of regulating epigenetic modifications that play important roles in oncogenesis (**Figure 2D**). Many studies demonstrate that successful survival and colonization of bacteria in the host can be achieved by altering host epigenetic events (70, 71).

Gastric microbiota such as *H. pylori, Kytococcus sedentarius* and *Actinomyces oris* can induce gastric cancer through the induction of abnormal DNA methylation patterns (72–74). *H. pylori* infection promotes the demethylation of guanine nucleotide-binding protein subunits β-44 (GNB4), thereby activating carcinogenic pathways (73). An analysis shows that some intratumoral microbiota in HCC tissues are intimately associated with the differential expression of many methylation-related genes (75). Increased levels of intratumoral *F. nucleatum* in CRC have been shown to be linked to hypermethylation of the promoter CpG island of the *CDKN2A* gene (76), a tumor suppressor gene. Moreover, *F. nucleatum* and *Hungatella hathewayi* (*H. hathewayi*) can increase the expression of DNA methyltransferases and then promotes the hypermethylation of tumor suppressor genes (TSGs), thereby enhancing CRC progression (77). *F. nucleatum* can also indirectly influence histone modification by regulating long noncoding RNA (lncRNA) (78). *F. nucleatum* increases the level of lncRNA enolase1-intronic transcript 1 (ENO1-IT1). The upregulated ENO1-IT1 can activate KAT7 histone acetyltransferase and then altered the histone modifiers on its target genes. This process subsequently promotes glycolysis and exacerbates CRC (78). *nucleatum* can also indirectly induce DNA hypermethylation by stimulating the production of reactive oxygen species (ROS), resulting in DNA damage and repair (79). Additionally, some viruses have been found to affect epigenetic regulation of their host cells (80, 81). Pietropaolo et al. identified seven oncoviruses, including HBV, KSHV, MCPyV, hepatitis C virus (HCV), high-risk HPV (HR-HPVs), Epstein-Barr virus (EBV), and T-lymphotropic virus 1 (HTLV-1) (81). They can alter DNA methylation, histone modification, ncRNA expression and chromatin remodeling, thereby playing important roles in carcinogenesis (81).

For example, HBV can induce m^6^A modification to influence virus replication and immune escape, thereby contributing to carcinogenesis (82, 83).

### Interference with cancer-related signaling pathways

Intratumoral microbiota can regulate tumor progression through many signaling pathways (**Figure 2E**). Some examples of how specific bacteria and their products affect cancer-related signaling pathways are shown below.

*H. pylori* produces a special protein, derived from the cytotoxin-associated gene (CagA), that can be translocated into host cells, triggering a cascade of downstream effects (84). Unmodified CagA in host cells directly interacts with polarity-regulating kinase partitioning-defective 1b (PAR1b), a kinase regulating cell polarity, and suppresses its activity. This interaction leads to the loss of cell polarity, a hallmark that precedes the development of dysplasia and carcinoma (85). Non-phosphorylated CagA also stimulates β-catenin activity and then increase the expression of oncogenes, contributing to the induction of gastric dysplasia and adenocarcinomas (86). Moreover, CagA can bind to the transmembrane protein junction adhesion molecule-A (JAM-A) and the epithelial tight-junction scaffolding protein (ZO1), thereby disrupting the assembly of tight junctions at bacterial attachment sites (87). CagA in some *H. pylori* strains can activate NF-κB activity, which in turn leads to an increase in interleukin-8 (IL-8) expression (88). This upregulation promotes neutrophil infiltration in the gastric mucosa.

*Salmonella typhi* secrets a pathogenic protein called AvrA, which has been found to elevate colon tumor incidence and promote tumor metastasis to proximal colon. Further experiments demonstrate that AvrA functions by increasing β-catenin levels and activating β-catenin signaling pathways (89). In another study, *Salmonella typhimurium*, when intravenously injected into colon tumors, can increase necrosis factor alpha (TNF-α) and IL-1β expression, as well as the associated downstream signals, leading to anti-tumor effects (90). *F. nucleatum* encodes an adhesion molecule known as FadA, which can bind to E-cadherin and activate β-catenin signaling. This interaction regulates oncogenic responses, contributing to tumor progression (91). *ETBF* secretes *B. fragilis* toxin (BFT) to induce E-cadherin cleavage, leading to the activation of β-catenin signaling (92). *Propionibacterium acnes (P. acnes)* is identified as an important intratumoral strain implicated in epithelial ovarian cancer (EOC) development (93). *P. acnes* is determined to increase the levels of inflammatory factors, including TNF-α and IL-1β, thereby enhancing inflammatory responses (93). Further experiments indicate that the inflammation caused by *P. acnes* can upregulate factors in the hedgehog (Hh) signaling pathway, thereby causing EOC progression (93). John et al. identified a series of intratumoral microbiota that are enriched in different types of papillary thyroid carcinoma (PTC), and these microbes may affect PTC through various pathways (94). Several fungal microbes are involved in classical PTC by regulating the phosphatidylinositol 3-kinase (PI3K)/protein kinase B (Akt), rat sarcoma (RAS) and B-Raf proto-oncogene, serine/threonine (BRAF) kinase pathways (94). In follicular variant PTC, the p53, BRAF kinase, mitogen-activated protein kinase (MAPK), and RAS signaling pathways may be involved. The BRAF kinase and MAPK signaling pathways are associated with Tall Cell PTC development (94). In general, the complex signaling networks are crucial in how intratumoral microbiota regulate tumorigenesis.

In conclusion, intratumoral microbiota may modulate the development of cancer through diverse mechanisms. However, it should be noted that while significant findings have been made, there might be other underlying mechanisms that require further exploration. Additionally, different studies may present varying viewpoints on this complex relationship. Ongoing collaboration between researchers and clinicians is essential for translating these insights into effective cancer prevention and therapeutic strategies.

## Roles of intratumoral microbiota in tumor metastasis

**Figure 3:**
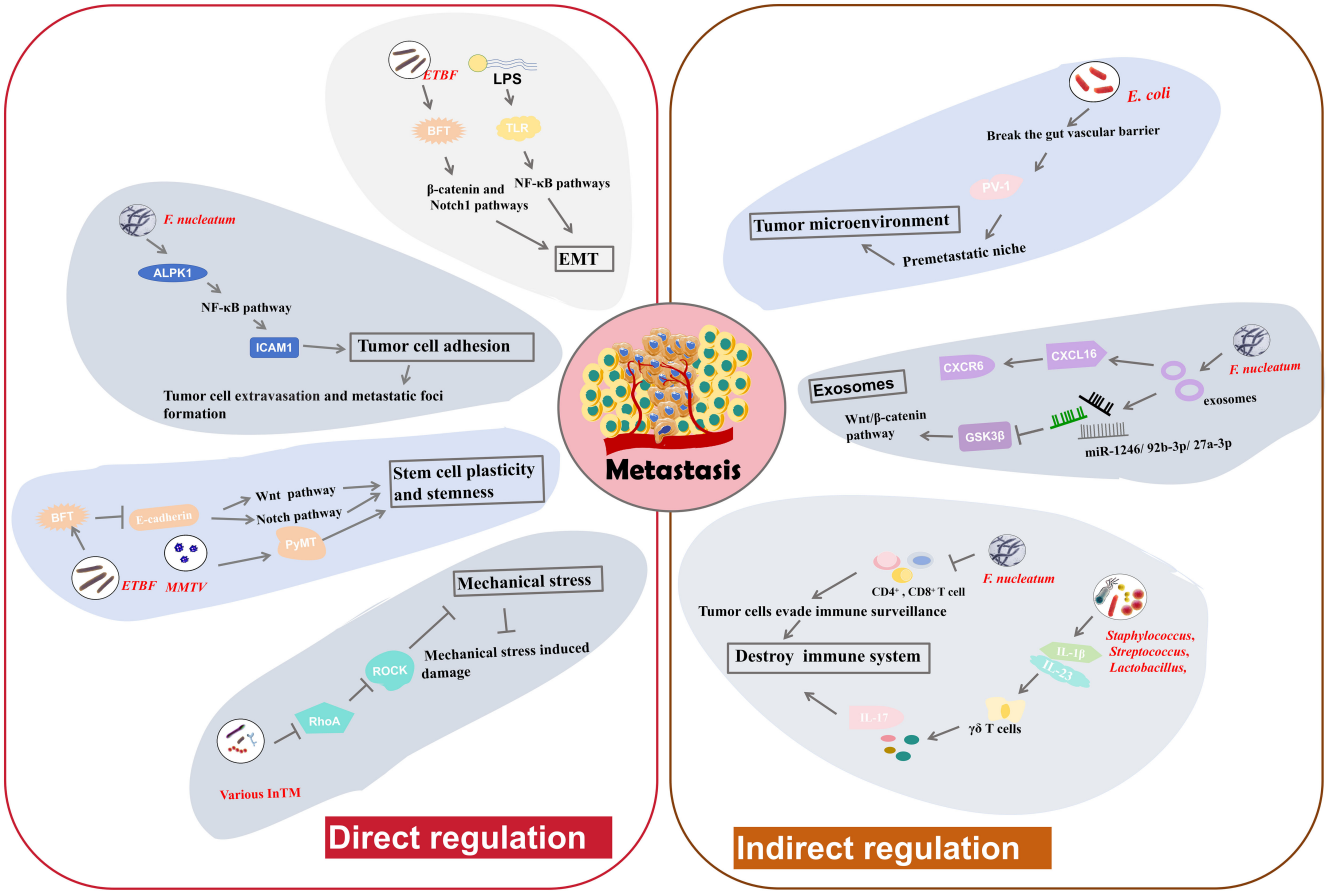
The regulatory mechanisms of intratumoral microbiota in tumor metastasis. Intratumoral microbiota may alter cancer cell-intrinsic properties such as cell adhesion, stem cell plasticity and stemness, mechanical stresses and EMT, to directly modulate tumor metastasis. Moreover, they can influence the host immune system and the tumor microenvironment to indirectly regulate tumor metastasis.

Tumor metastasis is an important factor in the deterioration of cancer and a major contributor to cancer-related mortality. Although the extensive effects of gut microbiota in tumor metastasis have been studied, the role of intratumoral microbiota in tumor metastasis is still an area of active research and exploration. Based on the current studies, we find that intratumoral bacteria may directly participate in tumor cell metastasis by regulating biological processes such as cell adhesion, stem cell plasticity and stemness, mechanical stresses and EMT (**Figure 3**). In addition, intratumoral microbiota may also indirectly affect tumor metastasis by influencing the host immune system, regulating the tumor microenvironment, and other pathways (**Figure 3**).

### Direct modulation in tumor cell properties

Adhesion molecules facilitate the strong cohesion between cells or with the ECM. Loss of adhesion enables tumor cell to detach from the primary tumor and invade into the bloodstream, potentially triggering anchorage-dependent cell death, known as anoikis (95). Main function of anoikis is to prevent abnormal cell growth or adhesion to irregular ECM, thus playing a vital role in maintaining tissue homeostasis. Resistance to anoikis may be critical in tumor metastasis, allowing tumor cells to survive and spread to distant organs through the circulatory system (96). *F. nucleatum* has been implicated in facilitating tumor cell adhesion and metastasis. Zhang et al. found that *F. nucleatum* could promote CRC cell adhesion to endothelial cells by increasing the expression of intercellular adhesion molecule 1 (ICAM1) (9), a transmembrane glycoprotein functioning in cell-cell adhesion and the inflammatory response (97). This increase in adhesion impels tumor cell to extravasate from the bloodstream and form metastatic foci (9). Alpha-kinase 1 (ALPK1) is a cytosolic recognition receptor for ADP-heptose (ADP-Hep) in Gram-negative bacterial (98). *F. nucleatum* upregulates ALPK1, leading to activation of NF-κB pathway (9), which in turn increases ICAM1 expression. Further studies indicate that high ALPK1/ICAM1 expression may suggest poor overall survival of CRC patients, indicating potential clinical significance (9). Therefore, *F. nucleatum* can facilitate CRC cell metastasis by regulating the ALPK1-NF-κB-ICAM1 pathway. This finding highlight the potential of targeting the ALPK1-NF-κB-ICAM1 pathway as a novel therapeutic strategy to resist CRC metastasis (9).

EMT is a process by which polarized epithelial cells acquire mesenchymal traits, becoming more migratory and invasive (4). This transition is a key event in the initiation of tumor metastasis. The adhesion between mesenchymal cells is very loose, allowing them to detach from the primary tumor, penetrate surrounding tissues, and migrate to distant sites in the body through the bloodstream or lymphatic system (4). Some intratumoral microbiota can induce EMT, facilitating metastasis. *B. fragilis* is a normal inhabitant of breast cancer tissues. BFT secreted by ETBF triggers epithelial hyperplasia (8). Research suggests that ETBF colonization in mammary ducts may induce EMT, thereby enhancing cancer growth and metastasis (8). The proposed mechanism involves the activation of the β-catenin and Notch1 pathways. BC cells exposed to BFT develop a heightened sensitivity to BFT, creating a “BFT memory”, that exerts long-standing promoting effects on tumor growth and metastasis (8). Lipopolysaccharide (LPS) is a necessary component of the outer wall of Gram negative bacteria. LPS is also able to induce EMT through the toll-like receptor (TLR)-NF-κB signaling pathways (99). Activation of these pathways induces inflammatory responses and promotes the expression of EMT-related genes, thereby enhancing tumor progression and metastasis (99). Slowicka et al. constructed a mouse model of colon cancer driven by EMT (100). In this model, tissue-resident microbiota can influence EMT processes, indicating the possible relationship between the presence of specific microbiota and tumor development through EMT (100). In summary, the ability of intratumoral microbiota to induce EMT through various mechanisms plays a pivotal role in cancer metastasis.

Cellular plasticity and stemness can help cells to transition between different states and to acquire new phenotypic characteristics. This ability to reprogram and alter cellular fate enable them to maintain homeostasis and facilitate tissue regeneration after damage (101). E-cadherin is a cell adhesion molecule that drives cell-cell connection, thereby enhancing tissue morphogenesis and embryonic development (101). Disruption of E-cadherin function, cells results in the loss of intercellular connections, potentially leading to cell dispersion, invasion, and metastasis. Clinically, normal levels of E-cadherin usually indicate a lower degree of malignancy and a better prognosis, whereas a negative E-cadherin status points to a higher malignancy grade and poorer outcomes (102). Sheetal Parida and colleagues found that BFT can destroy E-cadherin function, leading to the nuclear localization of β-catenin and the aggregation of Notch effector NICD in the nuclei of BC tissues (8). Subsequently, this process activates the Wnt and Notch pathways to increase cell stemness and cellular plasticity, subsequently leading to enhanced metastatic potential (8). The Mouse Mammary Tumor Virus (*MMTV*) induces the high expression of PyMT oncogene, alters cell plasticity and subsequently triggers the malignant proliferation of breast cells (27). The MMTV-PyMT pathway is widely used to construct breast tumor model. In a MMTV-PyMT model, the administration of certain tumor-resident microbiota into PyMT tumor cells can enrich cell stemness, thereby further promoting metastasis (27). These findings demonstrate that intratumoral microbiota can drive cellular plasticity and stemness to facilitate tumor metastasis.

Mechanical stress is also associated with tumor metastasis, as high mechanical stresses in the bloodstream, such as fluid shear stress, can damage circulating tumor cells (103, 104). Thereby, suppression of mechanical stress- induced damage is important for successful tumor metastasis and colonization at distant locations. Integrins, a family of cell adhesion molecules, can perceive mechanical stresses (105). They transmit following signals consisting of a cascade of RhoGTPase signaling and the Yap/Taz transcription factors, which are essential in tumor cell survival and the metastatic process (105). Some intratumoral microbiota may activate fluid shear stress signaling pathways to help tumor cells endure mechanical stresses and improve survival rates as they traverse the circulatory system. Fu et al. illustrated that various InTM, such as *S. xylosus*, *L. animalis*, *S. cuniculi*, and *S. sanguinis* can infiltrate into host tumor cells and facilitate their resistance to fluid shear stress in the circulatory system, thereby enhancing metastasis (27). These invaded tumor cells can transport the bacteria to metastatic sites (27). The bacterial invasion inhibits RhoA (a RhoGTPase) and Rho-associated protein kinase (ROCK) activity, thereby disassembling stress fiber and decreasing contractile forces. Downregulation of RhoA signaling pathway significantly suppresses the damage induced by mechanical stress, thereby enabling metastasis and the establishment of metastatic colonies (27). Therefore, InTM create a protective effect for circulating tumor cells to resist mechanical stress by regulating the RhoAGTPase-Rock-actin cytoskeleton organization pathway (27).

### Indirect modulation in extracellular physiological activities

In addition to the direct regulation pathways, intratumoral microbiota also facilitate tumor metastasis by modulating the immune reactions, gut vascular barrier and tumor exosome function.

The immune system serves as a vital barrier against tumor metastasis. Intratumoral microbiota can be identified by the immune system and then influence immune responses, such as T-cell-mediated immune responses. Intratumoral administration of *F. nucleatum* in BC has been confirmed to reduce the number of infiltrated CD4^+^ and CD8^+^ cells, which are typical immune related T cells, thereby allowing tumor cells to evade immune surveillance (7). This suppression in T-cell-mediated immune responses ultimately promote tumor progression and tumor cell metastasis (7). In a murine melanoma cancer model, antibiotic administration to reduce bacteria load causes a decrease in Tregs and an increase in conventional T cells, along with the activation of natural killer (NK) cells (106). These events significantly suppress the metastasis of melanoma cells to the lungs (106). Bacteria isolated from antibiotic-treated lungs also inhibit metastasis. Moreover, treatment with aerosolized *Lactobacillus rhamnosus* significantly enhances the immune responses against lung metastasis of melanoma cells (106). Lung-resident bacterial communities can induce inflammation related to lung cancer (24). They promote the generation of IL-1β and IL-23 by myeloid cells, and then induce proliferation of γδ T cells that facilitate formation of IL-17 and other inflammatory molecules, leading to inflammation that exacerbates tumor progression (24). In general, regulating immune responses is a key mechanism by which intratumoral microbiota can affect tumor metastasis.

The gut vascular barrier (GVB) refers to an anatomical structure that is critical for maintaining tissue integrity and preventing the dissemination of pathogenic bacteria communities through the gut to other organs. Intratumoral microbiota can impair this barrier and facilitate the transfer of tumor cells to the circulatory system, thereby promoting metastasis (45). *E. coli*, a common inhabitant of CRC, has been shown to break the GVB and spread from the gut to the liver. This bacterial dissemination facilitates the establishment of a premetastatic niche, a site characterized by an impaired immune system, and the recruitment of metastatic cells (45). The mechanism by which *E. coli* disrupts the GVB involves a virulence factor named PV-1. Elevated PV-1 levels in impaired GVB are positively correlated with increased bacterial dissemination from CRC tissue to liver (45). Thus, gut resident bacteria might promote metastasis by breaking the GVB via PV-1 (45). In another study, *Fusobacterium*, *Bacteroides*, *Selenomonas*, and *Prevotella* species that colonize CRC tumor cells have been implicated in promoting distal metastasis of primary colon cancer cells to the liver (107). This process may also involve GVB impairment. Antibiotic metronidazole treatment in mice with a colon cancer xenograft results in decreased *Fusobacterium* abundance and inhibited tumor growth (107). These findings suggest that targeting specific bacteria associated with the disruption of GVB could be a viable strategy to limit tumor metastasis.

Exosomes are vesicles derived from different types of cells that encapsulate a variety of substances, including proteins, lipids, nucleic acids, and so on (108). They play significant roles in intercellular communication. Tumor-derived exosomes are particularly effective in promoting tumor metastasis (108). They can be taken up by recipient cells to prepare the premetastatic niche, predict metastatic probability and determine the metastatic sites in different organs (109). Intratumoral microbiota can influence the production and content of exosomes, thereby affecting tumor metastasis. Exosomes can also carry intratumoral microbiota or microbial communities, helping them influence remote sites or prepare the pre-metastatic niche. *F. nucleatum* in CRC cells can secret special exosomes, termed Fn-Ex (37). CRC cell lines treated by Fn-Ex can alter the cell morphology and enhance cell migration. Some miRNAs (miR-1246/92b-3p/27a-3p) enriched in Fn-Exs can directly suppress glycogen synthase kinase-3β (GSK3β) expression and activate Wnt/β-catenin pathway (37), thereby promoting CRC cell migration, and ultimately contributing to tumor metastasis. Moreover, Fn-Exs also contain high levels of a chemokine, C-X-C motif chemokine 16 (CXCL16). CXCL16 facilitate tumor cell migration by interacting with its receptor CXCR6. These observations indicate that exosomes derived from CRC cells infected with *F. nucleatum* can stimulate tumor metastasis via the miR-1246/92b-3p/27a-3p-GSK3β-Wnt/β-catenin pathway or the CXCL16-CXCR6 pathway (37). All findings suggest that adjacent tumor cells may initiate metastasis using paracrine exosome secreted by bacteria-infected tumor cells.

Apart from these direct or indirect mechanisms by which intratumoral bacteria regulate metastasis, more efforts are still required to discover other pathways and their roles in tumor progression and metastasis need in-depth exploration.

## Clinical perspective of intratumoral microbiota

Studies have confirmed that 90% of cancer patients die from recurrence or metastasis (110, 111). As an important factor affecting metastasis, intratumoral microbiota can be used to predict survival rate of cancer patients. In a pancreatic cancer study, higher microbial diversity was revealed to indicate prolonged overall survival in pancreatic cancer patients (21). Research on nasopharyngeal carcinoma determined that the intratumoral microbiota were able to distinguish the degree of tumor deterioration (34), thereby aiding in treatment decisions and prognostic assessments. Moreover, in various types of cancers, intratumoral microbiota have been shown to be associated with the diagnosis or prognosis of metastasis (40, 41). These findings provide support for the development of treatment plans and surveillance strategies so as to manage the risk of metastasis more effectively.

Intratumoral microbiota may also be therapeutic targets to disrupt tumor growth and metastasis. Potential therapeutic strategies might consist of changing the microbiota composition to create a detrimental environment for tumor cells and introducing beneficial microorganisms to inhibit tumor progression. Microbial intervention strategies for cancer has long been established. In 1893, doctor William Coley used inactivated bacterial mixture (Coley’s toxin) to treat cancer and achieved success with 896 documented cases (23, 112). Bloch et al. discovered the suppressive effect of bacteriophages in malignant tumor growth in 1940 (113). To treat cancer using microorganism has attracted wide attention. In 1981, the first anticancer vaccine, HBV vaccine, was developed offering protection to numerous individuals at risk for hepatocellular carcinoma (HCC) (113). Subsequently, numerous bacterial vaccines have been developed to target microbe associated with cancers, potentially preventing tumorigenesis.

The ability of intracellular and extracellular shuttle enables intratumoral microbiota to act as engineering bacteria, delivering drugs to tumor sites. Particularly, these bacteria can specifically target tumor cells, thereby reducing collateral damage to healthy tissues. Moreover, intratumoral microbiota are commensal, addressing the safety and pathogenic concerns associated with the use of exogenous bacteria. This approach may be particularly effective in targeting metastatic cells, potentially decreasing the cancer recurrence rate.

Overall, intratumoral microbiota have high clinical value and necessitates comprehensive and rigorous investigations.

## Limitations

Despite these perspectives, some limitations are to be resolved for future application. First, there is a lack of systematic and precise experimental methods to thoroughly investigate the mechanisms of intratumoral microbiota. The methods for precisely manipulating intratumoral microbiota functions, monitoring their spatiotemporal dynamics, and effectively altering the bacterial genome for mechanism exploration remain undefined. Therefore, it is imperative to establish appropriate and effective experimental methods. Second, intratumoral microbiota are present in low abundance in cancer tissues, only 0.1-10% of cells containing bacteria (10, 27). This adds to the difficulty in every experimental procedure. Due to the low abundance, highly sensitive and specific detection methods are required. Developing better methods for enriching and cultivating intratumoral bacteria might help in isolating specific strains for further study. Third, the composition of intratumoral microbiota is very complex and dynamic in different tumors, but some studies lack detailed information on the composition of strains. This may hinder the replication of experiments and lead to inconsistent results, introducing bias in scientific understanding and confusion among readers. To address this, standardize experimental protocols are needed to minimize variability and improve reproducibility. Fourth, there are still many unknown relationships between tumor microbiota and tumor metastasis. To gain a deeper understanding on the mechanisms of intratumoral microbiota regulating tumor metastasis, more investigations are required by integrating knowledge from multiple disciplines, including microbiology, oncology, immunology, and computational biology. Longitudinal studies that track the evolution of intratumoral microbiota can supply valuable insights into the potential mechanisms of metastasis.

Altogether, overcoming the limitations will require innovative experimental approaches, improvement on repeatability and standardization, and multidisciplinary research. Solving these problems will help to bridge the gaps between basic research and clinical applications. Recently, researchers have been trying to integrate different technologies such as multi omics, spatial transcriptomics, and single-cell sequencing to investigate the multiple roles of intratumoral microbiota in carcinogenesis. The comprehensive understanding will pave the way for innovative treatment strategies in cancer diagnosis and treatment.

## Conclusion

All findings revealed that intratumoral microbiota can regulate tumor metastasis by modulating the host immune system, the tumor microenvironment, cell adhesion, stem cell plasticity and stemness, mechanical stresses and the EMT. However, in-depth investigations are still needed to uncover more underlying mechanisms, thereby providing theoretical support for innovative therapeutic strategies and clinical treatments for tumors.
